# Biaxial strain effect induced electronic structure alternation and trimeron recombination in Fe_3_O_4_

**DOI:** 10.1038/srep43403

**Published:** 2017-02-23

**Authors:** Xiang Liu, Li Yin, Wenbo Mi

**Affiliations:** 1 Tianjin Key Laboratory of Low Dimensional Materials Physics and Preparation Technology, Faculty of Science, Tianjin University, Tianjin 300072, China

## Abstract

The Verwey transition in Fe_3_O_4_ is the first metal-insulator transition caused by charge ordering. However, the physical mechanism and influence factors of Verwey transition are still debated. Herewith, the strain effects on the electronic structure of low-temperature phase (LTP) Fe_3_O_4_ with *P*2/*c* and *Cc* symmetries are investigated by first-principles calculations. LTP Fe_3_O_4_ with each space group has a critical strain. With *P*2/*c*, Fe_3_O_4_ is sensitive to the compressive strain, but it is sensitive to tensile strain for *Cc*. In the critical region, the band gap of LTP Fe_3_O_4_ with both two symmetries linearly increases with strain. When strain exceeds the critical value, DOS of spin-down *t*_2*g*_ electron at Fe(*B*4) with *P*2/*c* and Fe(*B*42) with *Cc* changes between *d*_*x*_2_-y_2 and *d*_*xz*_ + *d*_*yz*_. The trimerons appear in *Cc* can be affected by strain. With a compressive strain, the correlation of trimeron along *x* and *y* axes is strengthened, but broken along the face diagonal of Fe_*B*4_O_4_, which is opposite at the tensile strains. The results suggest that the electronic structure of Fe_3_O_4_ is tunable by strain. The narrower or wider band gap implies a lower or higher transition temperature than its bulk without strains, which also gives a glimpse of the origin of charge-orbital ordering in Fe_3_O_4_.

As an ancient magnet, Fe_3_O_4_ has been used as compass with a history about 3000 years^1^. With more in-depth understanding of Fe_3_O_4_, its novel properties including half-metallicity and high Curie temperature of about 860 K have potential applications in spintronic devices^2^,^3^. At ambient conditions, the high-temperature phase Fe_3_O_4_ has a face-center cubic lattice with a 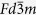 symmetry. As a mixed-valence iron oxide with an inverse spinel lattice, Fe_3_O_4_ is formally written as Fe_*A*_^3+^ [Fe^2+^Fe^3+^]_*B*_O_4_^4^. Two Fe_*B*_ atoms have one spin-down *t*_2*g*_ electron in 3*d* orbits^5^. Rapid hop of the electron between two neighbor Fe_*B*_ forms the conductive mechanism of Fe_3_O_4_.

In 1939, Verwey found that the conductivity of Fe_3_O_4_ drops about two orders by cooling down to 125 K^6^. The lattice symmetry transforms from cubic to monoclinic simultaneously. The first-order approximation given by Verwey is an order-disorder transition of charge distribution at Fe_*B*_^7^. The lattice structure of low-temperature phase (LTP) Fe_3_O_4_ once puzzled us. Gradually, the lattice structure of LTP Fe_3_O_4_ was clarified by X-ray diffraction, Raman and infrared spectroscopy in recent thirty years^1^,^8^,^9^,^10^,^11^. Below *T*_V_, the lattice is a suppercell of 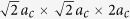 (*a*_*c*_ is the cubic lattice constant) with *Cc* symmetry. The charge ordering has been observed by Magnetic Compton scattering^12^, resonant multiwave X-ray diffraction^13^ and selected area electron diffraction^14^. With the *P*2/*c*^1^,^3^,^15^ or *Cc* space group^16^,^17^,^18^, some theoretical calculations on the charge-orbital distribution give the results that is consistent with the experiments, which describe the ionic distribution, complex charge-orbital ordering pattern and ferroelectricity.

Recently, Senn *et al*.^18^,^19^,^20^ proposed a new type of quasiparticle named as “trimeron” by both the experimental and theoretical results, where an anomalous shortage of the distance between Fe^2+^ and Fe^3+^ appears. The minority-spin *t*_2*g*_ electron is delocalized in a polaron that is composed of one Fe^2+^ donor and two Fe^3+^ acceptors^18^,^19^,^20^. Owing to the weak interactions, trimeron can be regarded as an orbital molecule, where three Fe ions locally coupled within an orbital ordered solid state. The trimeron model provides a new idea for understanding the Verwey transition. However, the case at a lattice strain may be different. High quality Fe_3_O_4_ samples grown on SrTiO_3_^21^ and Co_2_TiO_4_^22^ have been investigated, where the transition temperature shows a significant difference of about 10 K. So the strain may play an important role in the transition of Fe_3_O_4_, which should be studied in details.

In perovskite oxides (*AB*O_3_), *B* site is at the center of O octahedra^23^ and covalently bonding with the nearest O atoms^24^. Some previous results show that the tilting or rotation of the O-octahedra has an influence on the band gap of perovskite^24^,^25^. It is well known that the change of bond angle or bond length modifies the crystal field and band structure. Borisevich *et al*.^25^ indicate that the enlarged Fe-O-Fe angle and a higher symmetry can reduce the band gap of BiFeO_3_. By doping a ion with a larger atomic radium at *B*-site, Jiang *et al*. successfully tuned the band gap in CaFeO_3_^26^.

In order to investigate the strain effect on the charge-orbital ordering and electronic structure of LTP Fe_3_O_4_, the first-principles calculations are carried out on LTP Fe_3_O_4_ with *P*2/*c* and *Cc*. It is found that the orbital ordering and band structure of LTP Fe_3_O_4_ can be tuned by the external strain. The band gap of Fe_3_O_4_ with both two symmetries can be changed by a strain with a critical region, where the trimeron shows a complex relation with the external strain.

## Calculational Details

The electronic structures of the LTP Fe_3_O_4_ with structure (I) *P*2/*c*^1^ and structure (II) *Cc*^19^ are calculated by using the potential projector augmented wave method in Vienna Ab initio Simulation Package^27^,^28^. The calculations are based on the generalized gradient approximation plus on-site Column interaction (GGA + *U*)^29^,^30^,^31^. The energy cutoff is 400 eV. The Monkhorst-Pack grid of *k*-points for structure (I) is 6 × 6 × 2 and that for structure (II) is 3 × 3 × 2. The on-site Column interaction parameter *U* = 4.5 eV and on-site exchange interaction parameter *J* = 0.89 eV for all the Fe ions are used in the two structures^16^. The lattice constants and atomic positions in the two structures are used as that in refs 1 and 19, respectively. The same parameters except for *k*-points of 3 × 3 × 3 are used to calculate the high-temperature phase (HTP) Fe_3_O_4_ with structure (III) 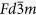 symmetry.

The stress is defined by the change of lattice constants as *S* = (*a*_*s*_ − *a*)/*a* × 100%, where *S, a* and *a*_*s*_ represents the strain, lattice constant without and with strain, respectively. Biaxial lattice strain is applied by fixing the in-plane lattice constants (*a* and *b*) and relaxing *z* direction throughout the calculations. The tensile and compressive strains are defined as *S* > 0 or *S* < 0. In order to clarify the strain effects on the charge-orbital ordering, the structural optimization for structures (I) and (II) with lattice constants are carried out, where the atomic positions are fully relaxed. Then, the lattice strain is taken into considerations. The structure optimization will stop until the total energy change is less than 10^−5^ eV and the Hellman–Feynman forces of optimized structure fall below 10^−2^ eV/Å.

## Results and Discussions

### Electronic & lattice structure with *P*2/*c* symmetry

**Figure 1:**
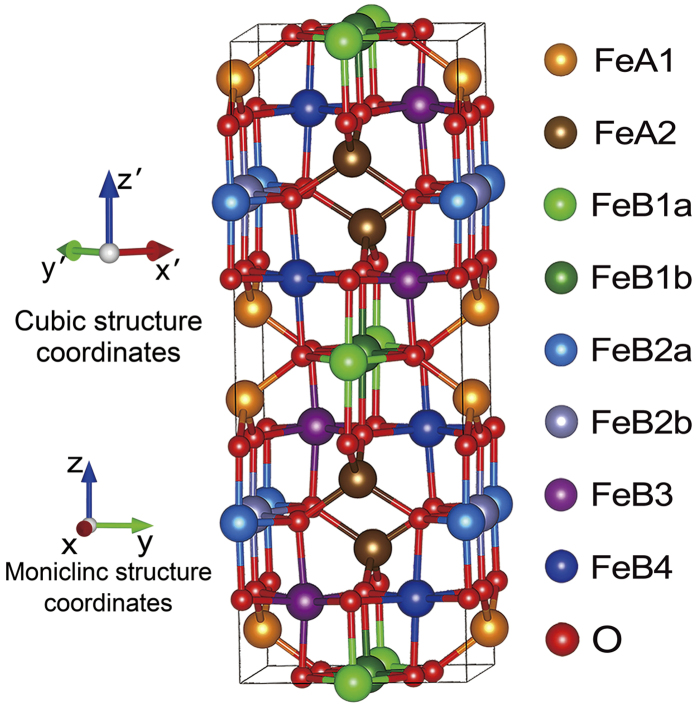
The lattice structure of Fe_3_O_4_ with *P*2/*c* symmetry. The equivalent atom sites of Fe ions have the same color. The monoclinic coordinate is rotated from cubic coordinate around *z* axis by 135°.

**Figure 2:**
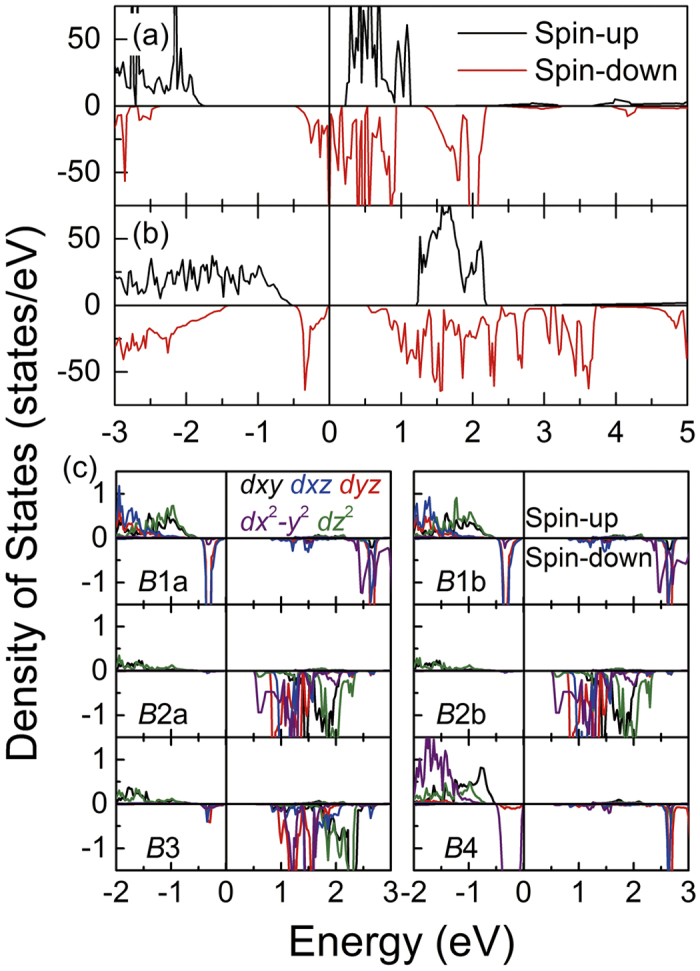
The total DOS of Fe_3_O_4_ with (**a**) 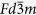 and (**b**) *P*2/*c* space group. The PDOS of different Fe_*B*_ sites in structure (I) projected on 3*d* orbits is shown in (**c**).

In Fig. 1, the unique equivalent site of Fe_*B*_ in structure (III) breaks into Fe(*B*1*a*), Fe(*B*1*b*), Fe(*B*2*a*), Fe(*B*2*b*), Fe(*B*3) and Fe(*B*4) in structure (I) as the symmetry reduces^1^,^3^. The coordinate system of monoclinic structure rotates by 135° around *z* axis^3^. Figure 2 shows the electronic structure of structure (I) and (III). HTP Fe_3_O_4_ shows a half metallic characteristic, where the spin-down states near Fermi level comes from the extra minority electron of Fe_*B*_ *t*_2*g*_ orbits^2^,^5^. In Fig. 2(b), the band gap of structure (I) is opened by 0.51 eV at Fermi level, which is a bit larger than the spectroscopic 0.14 eV^10^. Figure 2(c) shows the partial DOS at different Fe_*B*_ sites. The minority electron of Fe_*B*_ is localized at Fe(*B*1a), Fe(*B*1b) and Fe(*B*4), which is consistent with previous results^1^,^3^,^16^. These results suggest that Fe(*B*1a), Fe(*B*1b) and Fe(*B*4) are Fe^2+^ and Fe(*B*2a), Fe(*B*2b) and Fe(*B*3) are Fe^3+^. In Table 1, the bond-valence sum (BVS) of Fe_*B*_ is in well agreement with DOS. Herewith, the BVS expression is 
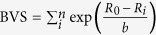
, where *R*_0_ is the bond-valence parameter^32^. For Fe^2+^ and Fe^3+^, *R*_0_ is 1.734 and 1.759, respectively. *R*_*i*_ refers to the *i*^*th*^ bond length and *b* is a constant of 0.37 Å^32^.

**Figure 3:**
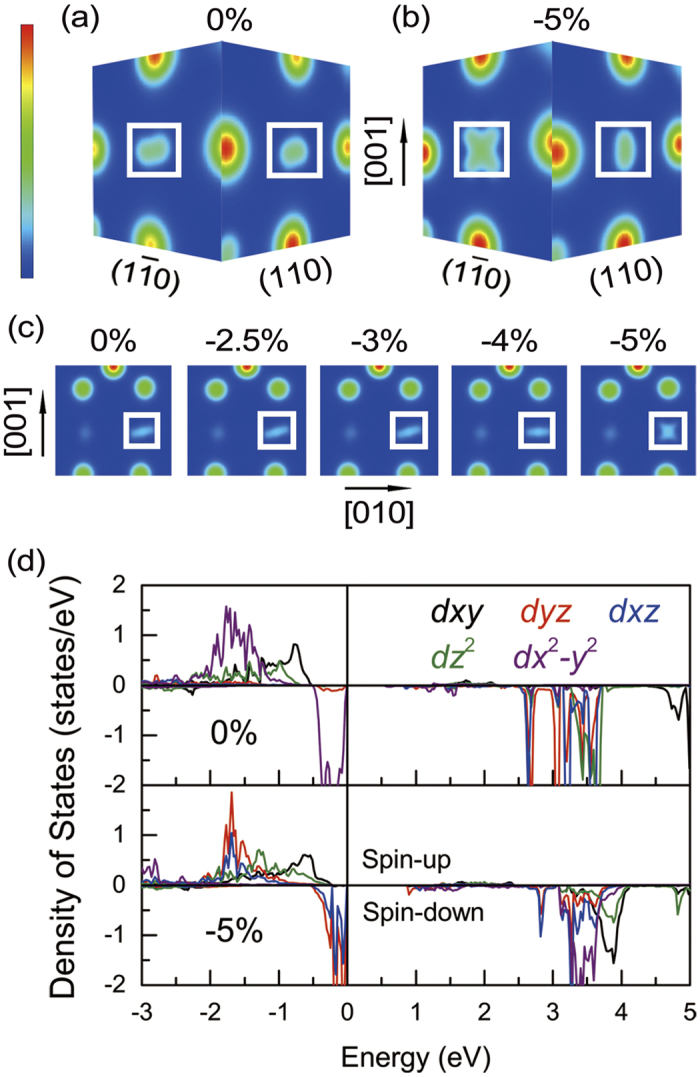
Charge density of *B*4*t*_2*g*_↓ plotted on 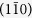 and (110) planes in structure (I) with (**a**) 0% and (**b**) −5% lattice strain are shown, respectively. Charge density of *B*4*t*_2*g*_↓ plotted on (100) plane for 0%, −2.5%, −3%, −4% and −5% lattice strain are showing in (**c**). The atom shown in white square is Fe (*B*4). The PDOS of Fe(*B*4) plotted on 3*d* orbits with 0% strain (upper panel) and −5% (lower panel) are shown in (**d**).

In order to investigate the strain effects, the strain of −5%, −2.5%, 0%, +2.5% and +5% are set. In Fig. 3, the orbit of spin-down *t*_2*g*_ electron at Fe(*B*4) (*B*4*t*_2*g*_↓) is almost in the *xy* plane at *S* = −2.5%, 0%, +2.5% and +5%. At *S* = −5%, the *B*4*t*_2*g*_↓ orbit shows a different style from others. In Fig. 3(a) and (b), the charge density of *B*4*t*_2*g*_↓ are projected onto 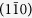 and (110) plan at *S* = 0% and −5%. At *S* = 0%, the charge density of *B*4*t*_2*g*_↓ plotted on both planes shows ellipsoidal shape. At *S* = −5%, the charge density of the electron plotted on (110) plane still shows ellipsoidal shape, but the charge density plotted on 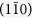 plane shows a flower shape. This phenomena manifests that the *B*4*t*_2*g*_↓ orbit lies in the 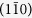 plane at *S* = −5%. In order to figure out the critical strain, the electronic structure is also calculated at *S* = −3% and −4%. Figure 3(c) shows the *B*4*t*_2*g*_↓ charge density projected onto (100) plane with a strain from 0% to −5%. The *B*4*t*_2*g*_↓ orbit still lies in the *xy* plane until the strain decreases to −5%. Therefore, the compressive strain of −5% is the critical value for *P*2/*c* symmetry. Meanwhile, DOS of 3*d* orbits of Fe(*B*4) also shows the same change. In Fig. 3(d), at *S* = −5%, the *B*4*t*_2*g*_↓ orbit changes from *d*_*x*_2_-*y*_2 to *d*_*yz*_ + *d*_*xz*_ by comparing the DOS at *S* = 0%. Actually, the *B*4*t*_2*g*_↓ orbit changes from *d*_*xy*_ to *d*_*yz*_ in HTP Fe_3_O_4_ coordinate system because of the rotation of coordinate^3^.

**Figure 4:**
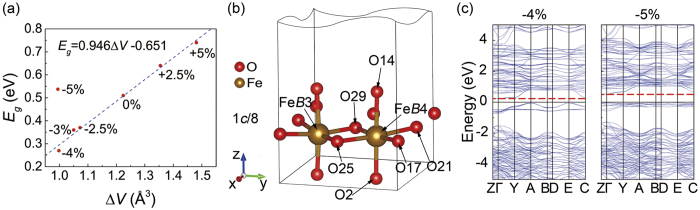
(**a**) The band gap *E*_*g*_ with the average FeO_6_ volume difference at different strains. (**b**) The local structure of Fe(*B*4) under *P*2/*c* symmetry. (**c**) The band structure of structure (I) with −4% and −5% compressive strain in left and right panel, respectively.

Since the conductivity of Fe_3_O_4_ is related to Fe*Bt*_2*g*_↓ and *B*O_6_ distortion, it is necessary to investigate the O-octahedral distortion at different Fe_*B*_ sites. In Fig. 4(a), the band gap shows a positive correlation with the increased Δ*V* except for *S* = −5%. Herewith, Δ*V* is the average volume difference between Fe^2+^O_6_ and Fe^3+^O_6_, *E*_*g*_ is the energy gap near Fermi level. By linear fitting *E*_*g*_ at different strains, we get *E*_*g*_ = 0.946Δ*V* − 0.651, where *E*_*g*_ at a compressive strain of −5% is not included. Quantitatively, the volume of FeO_6_ shows the magnitude of its crystal field. Since Fe^3+^ has a stronger interaction with surrounded O^2−^ than Fe^2+^, the volume of Fe^3+^ O_6_ is smaller than that of Fe^2+^O_6_, but the electrostatic potential is higher than that of Fe^2+^O_6_. The results mean that the larger volume difference is, the more energy is needed when the electron hopes between Fe^2+^ and Fe^3+^. Therefore, as the tensile strain increases, the gap becomes larger. Simultaneously, the competition between the band gap and thermal activation energy has a relation with the metal-insulator transition (MIT) temperature. Therefore, the MIT temperature of Fe_3_O_4_ could be tuned by external strain. At a compressive (tensile) strain, the band gap becomes narrow (wide) and the MIT temperature of Fe_3_O_4_ becomes lower (higher).

However, the above demonstration on the relation between Δ*V* and *E*_*g*_ is not proper for the case at *S* = −5%. Therefore, the Fe-O bond length in FeO_6_ at Fe_*B*_ is further analyzed. Since the charge density of Fe(*B*4) shows an obvious change and all the Fe(*B*4) atoms are equivalent with the same ambient ionic conditions, in Fig. 4(b), Fe(*B*4) at 1*c*/8 height is selected as a candidate. O(17), O(21), O(25), O(29) and Fe(*B*4) are almost in horizontal plane and O(2)-Fe(B4)-O(14) is parallel to *z* axis. Table 2 lists the Fe(*B*4)-O distances at different strains. In the horizontal plane, at *S* ≥ −4%, the bond length of Fe(*B*4)-O(17) and Fe(*B*4)-O(21) are longer than that of Fe(*B*4)-O(25) and Fe(*B*4)-O(29). At *S* = −5%, the distances Fe(*B*4)-O(17) and Fe(*B*4)-O(29) become much longer. The bond lengths of Fe(*B*4)-O(21) and Fe(*B*4)-O(25) show an anomalous shortage, but the Fe-O bond length along *z* direction shows a sudden enlargement. At *S* = −5%, the length of O(2)-Fe(*B*4)-O(14) is about 0.15 Å longer than that at *S* = −4%. The above phenomenon shows that the strain can tune the mode of ionic distribution and crystal field. At a larger strain, the orbital ordering pattern becomes unstable. The outermost 3*d* electrons of Fe(*B*4) have a strong Column repulsive interaction with surrounded O^2−^ in horizontal plane. Owing to the change of the distribution of 3*d* orbits, the electrostatic energy can be partially released along the O(25)-Fe(*B*4)-O(21) direction. O^2−^ has been pushed away along *z* direction due to the electronic interaction. The Fe-O bond length distortion in horizontal plane also appears at Fe(*B*2) and Fe(*B*3). However, the Fe-O bond lengths at Fe(*B*1) do not change. Since the inversion centers and partial face centers are occupied by Fe(*B*1), the symmetry of Fe(*B*1) is higher than other Fe_*B*_ sites, where the ambience of Fe(*B*1) is more stable than other Fe_*B*_ sites. Therefore, the mode of ionic distribution does not change at Fe(*B*1).

In the band structure, the energy of spin-down conduction-band minimum at *S* = −5% is higher by about 0.28 eV than that at *S* = −4%. However, in Fig. 4(c), the valence-band maximum of Fe_*B*_ *t*_2*g*_↓ is still just below Fermi level. The compressive strain of −5% can change the structure of O-octahedra at Fe_*B*_ sites. Simultaneously, the Fe-O Column interaction can raise the conduction band energy. So, in Fig. 4(a), the band gap of structure (I) at *S* = −5% is larger by about 0.28 eV than that at *S* = −4%.

Furthermore, the nearest six Fe-Fe distances around different Fe_*B*_ sites are analyzed. Unlike Column’s law, the <Fe^2+^-Fe^3+^> distance shows an anomalous shortage, which is even less than the <Fe^2+^-Fe^2+^> distance at Fe(*B*1) without strain. The phenomenon is consistent with trimeron model. As the tensile strain is applied, the weak bond interaction between Fe^3+^-Fe^2+^-Fe^3+^ becomes tighter around Fe(*B*1). When the compressive strain is applied, the trimerons around Fe(*B*1) become weak. However, the distance between Fe(*B*4) and Fe(*B*3) becomes shorter than the Fe^2+^-Fe^2+^ distance around Fe(*B*4). So, a more complex structure of trimeron forms, which will be demonstrated in the next section.

### Electronic & lattice structure with *Cc* symmetry

**Figure 5:**
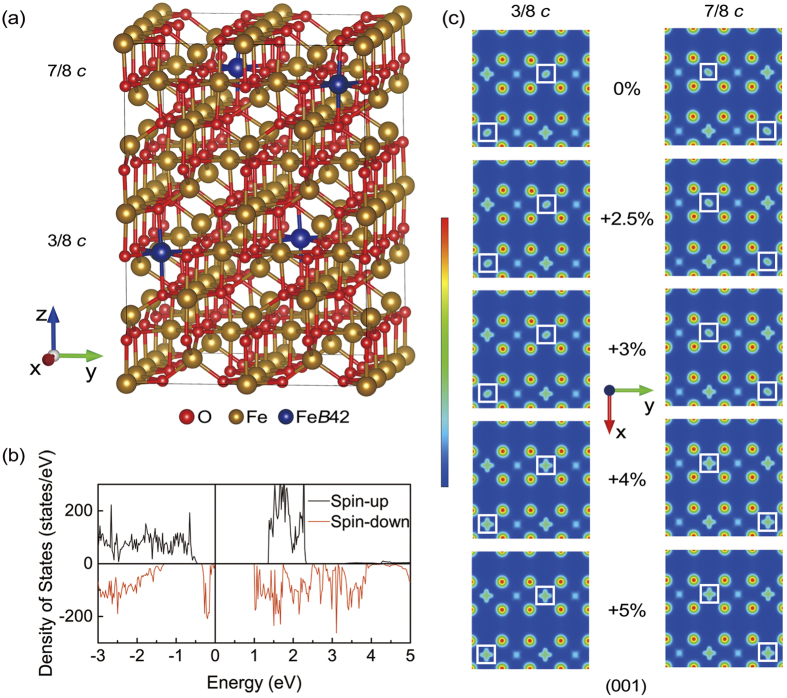
(**a**) The lattice structure under *Cc* symmetry. (**b**) Total DOS of structure (II). (**c**) Charge density map of spin-down electrons plotted on (001) plane at 3*c*/8 (left column) and 7/8 *c* (right column) with different strains. Fe(*B*42) is shown in the white square.

In Fig. 5, when the symmetry reduces to *Cc* space group, the LTP Fe_3_O_4_ lattice and the charge-orbital ordering pattern become more complex. The electronic structure of bulk without strain is firstly calculated. The band gap near Fermi level with and without structural optimization is about 1.0 and 0.7 eV, respectively. Figure 5(b) shows the total DOS of the optimized structure. The energy gap is larger than experimental result because the calculation is proceeded at 0 K. Then, the strain of −5%, −2.5%, +2.5% and +5% is applied to the *Cc* structure. Different from *P*2/*c* structure, the structure (II) is sensitive to tensile strain. So, we then calculated the electronic properties at *S* = +3% and +4% to figure out the critical value.

**Figure 6:**
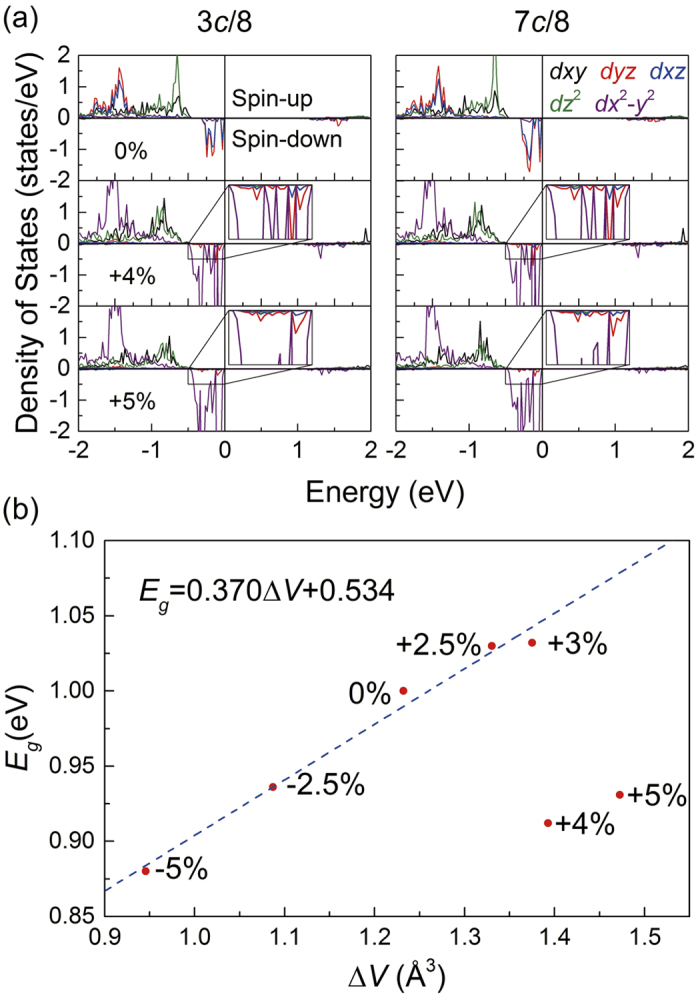
(**a**) PDOS of Fe(*B*42) plotted on 3*d* orbits with a strain of 0%, +4% and +5% at 3*c*/8 (left panel) and 7*c*/8 (right panel), respectively. The correspondent local magnifications are shown in the inset. (**b**) The dependency between the band gap *E*_*g*_ and the corresponding average FeO_6_ volume difference Δ*V* with different strains.

Figure 5(c) shows the spin-down charge density at a height of 3*c*/8 and 7*c*/8 as a tensile strain increases from 0% to +5%. At *S* < +4%, the Fe(*B*42)*t*_2*g*_↓ orbit [marked with white square in Fig. 5(c)] lies in the (110) plane at 3*c*/8 and lies in the 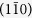 plane at 7*c*/8. When the tensile strain exceeds the critical value at *S* = +4% and +5%, the Fe(*B*42)*t*_2*g*_↓ orbit rotates into horizontal plane. Figure 5(a) shows the atom sites of Fe(*B*42), which is labeled in dark blue. In Fig. 6(a), at *S* = +4% and +5% the DOS of Fe(*B*42) shows that the orbits of those Fe atoms change from *d*_*yz*_ + *d*_*xz*_ to *d*_*x*_2_-*y*_2. The coordinate of structure (II) also rotates around *z* axis by 135° from cubic Fe_3_O_4_, which is consistent with structure (I). Therefore, the orbit actually changes from *d*_*xz*_ to *d*_*xy*_ at 3*c*/8, which changes from *d*_*yz*_ to *d*_*xy*_ at 7*c*/8 within cubic coordinate. In the inset of Fig. 6(a), by comparing the DOS at *S* = +4% and +5%, it is found that although the Fe(*B*42)*t*_2*g*_↓ orbit changes at *S* = +4%, it still has the residual states projected onto *d*_*yz*_. The residual states come from the out-of-plane slope of *d*_*x*_2_-*y*_2. Then, the relationship between Δ*V* and *E*_*g*_ in structure (II) is investigated. In Fig. 6, *E*_*g*_ shows a positive relation with the increased Δ*V* at *S* < +4%, which can be described as *E*_*g*_ = 0.370Δ*V* + 0.534. However, the linear fitting parameters both slope and intercept are quite different from structure (I) due to the different structure and charge-orbital ordering pattern.

**Figure 7:**
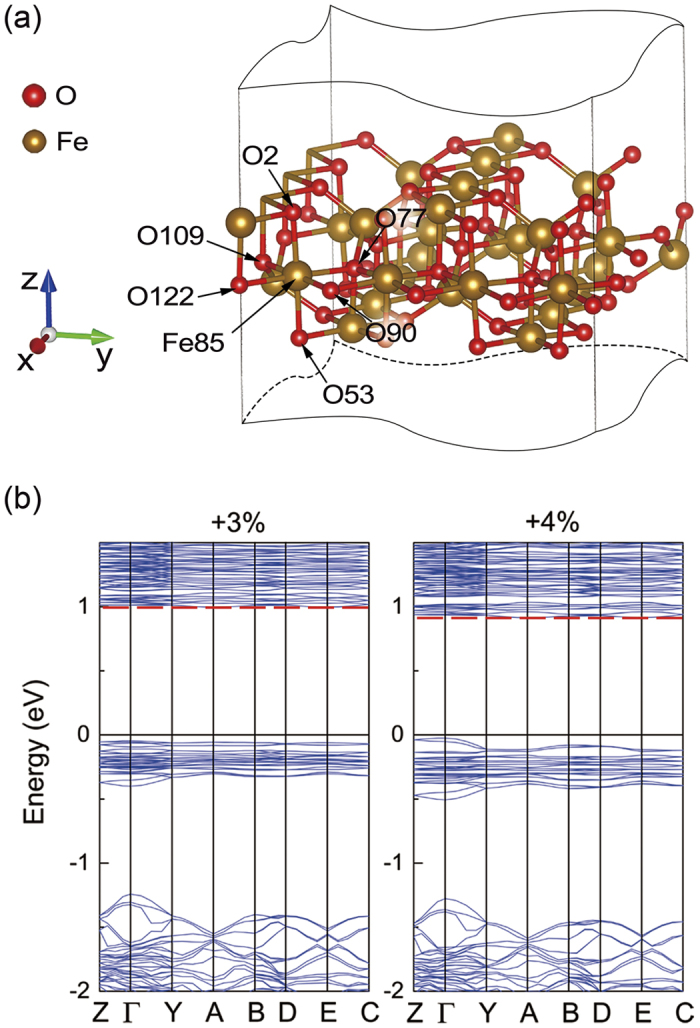
(**a**) The local structure of Fe(*B*42) under *Cc* symmetry. (**b**) The band structure under *Cc* symmetry with +3% and +4% compressive strain in left and right panel, respectively.

Since the orbital change at Fe(*B*42) is obvious, the Fe-O bond length and O-octahedra distortion at Fe(*B*42) are taken as an example, where Fe(85) (at 3*c*/8) is selected as a substitute for other equivalent Fe(*B*42) sites. Figure 7(a) shows the local structure of Fe(85). The O(77), O(90), O(109), O(122) and Fe(85) atoms are almost in horizontal plane. O(2)-Fe(85)-O(53) is almost parallel to *z* axis. Table 3 lists the Fe(85)-O bond lengths at different strains, revealing the reason for the orbital change at Fe(*B*42). The FeO_6_ distorts in horizontal plane at *S* = +4% and +5%. The two shortest bonds are along (110) direction and the two longest bonds are perpendicular to (110) direction. When +4% and +5% strain is applied, both the shortest and longest bond are coexistent in diagonals. The Fe-O bond length along *z* direction suddenly decreases by about 0.1 Å when the strain increases from +3% to +4%. The obvious Fe-O bond length distortion in *xy* plane also appears at Fe(*B*2b1), Fe(*B*31), Fe(*B*32), Fe(*B*34), Fe(*B*41), Fe(*B*43) and Fe(*B*44). Due to the tensile strain, the expansion of the equatorial plane of O-octahedra can release the electrostatic energy between the surrounded O^2−^ and outside electron of Fe^2+^. Correspondingly, the Column interaction along *z* direction can also be weakened by the transformation of Fe(*B*42)*t*_2*g*_↓ orbit, so the Fe-O bond length along *z* direction becomes shorter. Since the equatorial face can further expansion at *S* = +5%, more electrostatic energy can be released, where the Fe(*B*42)*t*_2*g*_↓ orbit becomes more parallel to the *xy* plane. In the inset of Fig. 6(a), at *S* = +5%, the residual *d*_*yz*_ states are less than that at *S* = +4%. In Fig. 7(b), the conduction-band minimum at *S* = +4% is lower than that at *S* = +3%, where the valence-band maximum is still just below Fermi level. As a result, the band gap becomes smaller as the strain increases.

**Figure 8:**
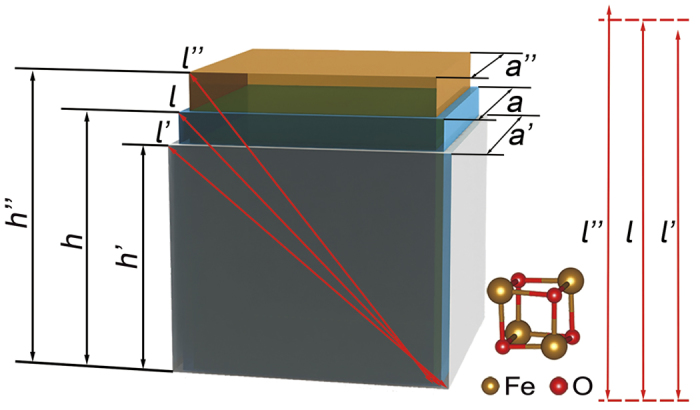
The sketch map shows the ideal model of deformation with equivalent volume. The Fe_*B*4_O_4_ models without strain, with tensile or compressive strain are colored with blue, grey and orange, respectively. *a, h* and *l* each respects the in plane, out of plane direction Fe-O bond length and the Fe-Fe distance along face diagonal direction. The length of *a*′ and *a*″ is (1 + 6%)*a* and (1 − 6%)*a*, respectively. The ratio of *a, a*′ and *a*″ is correspondent with the calculation results. The local structure of Fe_*B*4_O_4_ is also shown in the lower right corner.

Furthermore, the model of trimeron presented by Senn *et al*.^18^,^19^,^20^ is also observed in our calculations. It is found that the distribution of trimeron can be affected by external strain. When the strain increases from 0% to +5%, the Fe-Fe distance along *x* and *y* axis changes faster than that along the face diagonal direction of Fe_*B*4_O_4_. The Fe-Fe distance along diagonal direction even reduces with the increased strain at some Fe_*B*_ sites. The process of Fe_*B*4_O_4_ distortion is compared with an ideal model of equivalent volume deformation in tetragonal system. Figure 8 shows the sketch map of this ideal model, where *a, h* and *l* each respect the in-plane, out-of plane crystal edges and face diagonal. Lattice with tensile and compressive strain are superscripted with *′* and *′′*, respectively. The volume of this tetragonal *V* = *a*^2^*h*, so *h* = *V/a*^2^ and the face diagonal 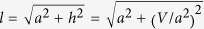. The different coefficient of *l* with respect to *a* is 
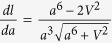
. At 0 < *a* < 3.367, 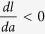. In our model, the case of 0 < *a* < 3 is considered. As a result, the length of face diagonal reduces with the increased lattice constant. So, the trimerons along *x* and *y* direction break down by the tensile strain, but the correlation of trimerons along the face diagonal are strengthened. When a compressive strain is applied, the Fe-Fe distance along *x* and *y* direction becomes short and the Fe-Fe distance along face diagonal elongates. The trimerons along *x* and *y* direction are strengthened, but the trimerons along the face diagonal directions break down due to the compressive strain.

## Conclusions

We have investigated the biaxial strain effects on the electronic structure of LTP Fe_3_O_4_ with *P*2/*c* and *Cc* space group by GGA + *U* method. When the strain on the two structures are below their critical region, the distortion of O-octahedra can change the electrical potential difference between the nearest ferric and ferrous ions. As a result, the band gap shows a positive linear correlation with the strain. The narrower or wider band gap implies a lower or higher transition temperature. When the strain is above the critical value namely *S* < −4% in structure (I), the orbit of Fe(*B*4)*t*_2*g*_↓ changes from *d*_*xy*_ to *d*_*yz*_ in HTP Fe_3_O_4_ coordinate and the energy of conduction-band minimum raises. In structure (II), at *S* ≥ +4%, the orbit of Fe(*B*42)*t*_2*g*_↓ changes from *d*_*yz*_ to *d*_*xy*_ in HTP Fe_3_O_4_ coordinate and the energy of conduction-band minimum reduces. The trimeron appears in both the structure (I) and (II). The distribution of trimeron can also be affected by strain. The trimerons along *x* and *y* axes get broken (strengthen) at a tensile (compressive) strain. However, the trimerons along face diagonal are broken (strengthened) at a compressive (tensile) strain. These results can be ascribed to the change of Fe-Fe distance when different strains are applied, which can be estimated by geometric calculations.

## Additional Information

**How to cite this article**: Liu, X. *et al*. Biaxial strain effect induced electronic structure alternation and trimeron recombination in Fe_3_O_4_. *Sci. Rep.* **7**, 43403; doi: 10.1038/srep43403 (2017).

**Publisher's note:** Springer Nature remains neutral with regard to jurisdictional claims in published maps and institutional affiliations.

## Figures and Tables

**Table 1:** The BVS calculation results of Fe_*B*_ in structure (I).

Atom site	BVS	<Fe_*B*_-O> (Å)
Fe(*B*1a)	2.238	2.101
Fe(*B*1b)	2.238	2.101
Fe(*B*2a)	2.942	2.023
Fe(*B*2b)	2.942	2.023
Fe(*B*3)	2.882	2.034
Fe(*B*4)	2.255	2.098

The bond-valence parameters for Fe^2+^-O^2−^ = 1.734 and Fe^3+^-O^2−^ = 1.759^32^. The average Fe_*B*_-O bond lengths in Fe_*B*_O_6_ at different Fe_*B*_ sites are also shown.

**Table 2:** The bond lengths (Å) between Fe(*B*4) at 1*c*/8 and surrounded O^2−^ in structure (I) with the strain changing from +5% to −5%.

StrainAtom site	+5%	+2.5%	0%	−2.5%	−3%	−4%	−5%
O(2)	2.0019	2.0163	2.0277	2.0360	2.0370	2.0413	2.1389
O(14)	2.0440	2.0664	2.0854	2.1052	2.1087	2.1144	2.1685
O(17)	**2.2159**	**2.1690**	**2.1314**	**2.0858**	**2.0777**	**2.0612**	**2.0618**
O(21)	**2.2170**	**2.1702**	**2.1317**	**2.0882**	**2.0802**	**2.0668**	1.9828
O(25)	2.1926	2.1461	2.1052	2.0598	2.0526	2.0390	1.9645
O(29)	2.1932	2.1456	2.1054	2.0576	2.0500	2.0323	**2.0566**

The two longest bond lengths in the *xy* plane are shown in bold.

**Table 3:** The nearest Fe-O bond lengths at Fe(85) with different stresses in structure (II).

Atom site	+5%	+4%	+3%	+2.5%	0%	−2.5%	−5%
O(2)	2.0252	2.0255	2.1162	2.1141	2.1331	2.1402	2.1426
O(53)	2.0323	2.0329	2.1019	2.1016	2.1199	2.1225	2.1234
O(77)	**2.2188**	**2.1966**	**2.1673**	**2.1552**	**2.1218**	**2.0800**	**2.0460**
O(90)	**2.2037**	**2.1797**	2.0850	2.0755	2.0405	2.0049	1.9752
O(109)	2.1968	2.1708	2.0969	2.0852	2.0466	2.0098	1.9787
O(122)	2.1986	2.1731	**2.1769**	**2.1658**	**2.1296**	**2.0853**	**2.0466**

The two longest bond lengths in the *xy* plane are shown in bold.
